# ECMO for Neonatal Sepsis in 2019

**DOI:** 10.3389/fped.2020.00050

**Published:** 2020-02-21

**Authors:** Warwick Wolf Butt, Roberto Chiletti

**Affiliations:** ^1^Paediatric Intensive Care Unit, Royal Children's Hospital, Melbourne, VIC, Australia; ^2^Murdoch Children's Research Institute, Melbourne, VIC, Australia; ^3^Department of Paediatrics, University of Melbourne, Melbourne, VIC, Australia

**Keywords:** sepsis, shock, ECMO, neonate, infection

## Abstract

Sepsis and septic shock in newborns causes mortality and morbidity depending on the organism and primary site. ECMO provides cardiorespiratory support to allow adequate organ perfusion during the time for antibiotics and source control surgery (if needed) to occur. ECMO mode and cannulation site vary depending on support required and local preference. Earlier and more aggressive use of ECMO can improve survival.

## Introduction

Sepsis secondary to a bacterial, a viral, or a fungal infection during the first 28 days of life remains a significant cause of mortality and long-term morbidity. Despite advances in neonatal care and maternal antibiotic prophylaxis for group B streptococcus (GBS), the incidence of neonatal sepsis remains high with 1–4 cases every 1,000 live births in the USA with mortality and long-term disability affecting 40% of neonates with sepsis (1–3). Risk factors for the development of sepsis in the neonatal period can be maternal (prolonged rupture of membranes, poor or no antenatal care, meconium-stained liquor, premature labor and chorioamnionitis, GBS colonization) and neonatal (prematurity, low birth weight, APGAR 5 min <5, male gender, resuscitation at birth, neutropenia, lack of enteral feeding, need for vascular catheters and mechanical ventilation) (4). While GBS and Escherichia Coli are the most common bacteria involved, viral sepsis (Herpes Simplex Virus, HSV) and fungal infections are responsible for increased mortality and neurological sequelae, especially in the premature group. Mortality rates for neonatal sepsis vary between 10 and 30% across studies based on gestational age (term vs. birth weight <1,000 g, 52% vs. 72%, respectively) and pathogen (up to 73% for systemic candidiasis) (1, 5–7).

Neonatal sepsis is a heterogeneous entity with different clinical presentations depending on the time of onset and is classified as early (within the first 72 h of life) and late-onset (beyond 72 h of life). These differences correlate with the physiological changes the myocardium and vascular system undergo during the first weeks of life.

The definition of sepsis in neonates is adapted from the pediatric population complicating further diagnosis and management of neonatal sepsis; in a retrospective review of term neonates, only 53% of the cases of culture-positive early-onset sepsis were diagnosed by the consensus definition (8, 9).

Extracorporeal mechanical oxygenation (ECMO) is routinely used around the world to support children and adults with respiratory and/or cardiovascular dysfunction with increasing numbers of children supported over the last three decades (10). The American College of Critical Care Medicine, in their latest edition of the neonatal sepsis guidelines, recommends ECMO for refractory shock as last tier intervention when medical management has failed (11). Despite early reluctance of the ECMO community in supporting adults and children with septic shock due to high morbidity and mortality, ECMO has been routinely utilized for the neonatal population with reported survival rate of up to 70% (12–16).

## Neonatal Cardiovascular Physiology vs. Children and Adolescents

**Figure 1:**
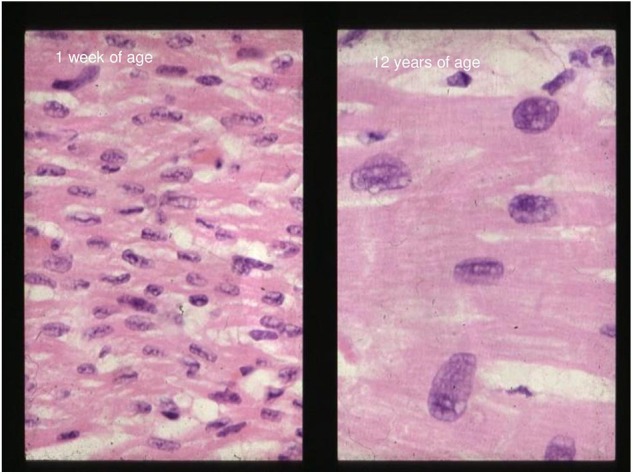
Stained myocardial section in a neonate and a 12 year old at same magnification.

The neonatal myocardium has functional and structural features that differ markedly from the heart of older children and adults (Table 1). The myocardium has less contractile protein per 100 grams of tissue than an older child's heart; only half of the tissue is composed of contractile elements while the remaining 50% is made of connective tissue, large nuclei, and mitochondria (see Figure 1). Therefore, the mass of the myocardium is reduced, as well as its compliance and contractile capacity and reserve. Equally important is the “disorganization” of the neonatal myocardium as compared to the well-organized sheets of muscle of the older child's heart. The reduced compliance of the heart leads to higher filling pressure and reduced pre-load augmentation (23–25). These factors limit the reserve capacity of the neonatal heart, making it highly dependent on heart rate and susceptible to negative inotropic drugs or acidosis. While adults hearts can double or triple their heart rate to maintain oxygen delivery (high output shock), the high baseline rate of neonates limits this compensatory mechanism. Furthermore, differently from adult hearts, cardiac function is highly dependent on the resting beta-adrenergic stimulation leading to a reduced response to beta-agonist agents, meaning higher doses are required in neonates to achieve the same effect.

**Table 1:** Pathophysiological differences for sepsis/septic shock by ages and ECMO survival.

**SEPSIS & SEPTIC SHOCK**	**Newborn**	**Child**	**Adult**
Physiological differences	↓ myocardial mass ↓ compliance and contractility High baseline HR with poor compensatory capacity ↓α-adrenergic receptors ↑ circulating catecholamines ↓ PMN recruitment and BM depletion ↓ phagocytic activity	Developmental transition from neonatal to adult features through first 5 years of life	Normal mass Normal compliance and contractility HR can double/triple to maintain DO2 Normal α-adrenergic receptors and circulating catecholamines PMN margination at infection site and inflammatory cascade activation
Incidence (USA)	1–5 cases per 1,000 live births	1 case per 1,000 person-year	13–78 cases per 100,000 person-year
Predominant cardiovascular status	↑PVR & ↓RV function ↓ LV function/CI	↓ LV function/CI & ↑or↓ SVR	↓ SVR and ↑ CI
Clinical features	PPHN & respiratory failure and/or cardiogenic shock	Cardiogenic shock and/or distributive shock	Distributive shock and/or cardiogenic shock
ECMO survival	50–77% (13, 16, 17)	31–74% (16, 18, 19)	22–78% (20–22)

*HR, heart rate; DO2, oxygen delivery; PMN, polymorphonuclear cells; BM, bone marrow; PVR, pulmonary vascular resistance; RV, right ventricle; LV, left ventricle; CI, cardiac index; SVR, systemic vascular resistance; PPHN, persistent pulmonary hypertension on the newborn*.

The neonatal myocardium expresses less alpha-adrenergic receptors which contribute to the reduced left ventricular function. To compensate for the reduced myocardial function, the concentration of circulating catecholamines is higher than in adults; therefore, the higher depressing effect in neonates on cardiac output by anesthetic agents. Calcium ion transport in the myocardial cells is fundamental to guarantee contraction and relaxation. Myocardial sarcolemma and T-tubular system is less well-developed and calcium entry into cells is compromised in the newborn. Also, neonatal myocytes have reduced mitochondrial calcium and rely mostly on extracellular calcium for contractility; volatile agents used in anesthesia, modulate calcium inflow and can lead to severe myocardial depression in neonates. Decreased kinetics of cytosol calcium contribute to delay in diastolic relaxation (26, 27).

During fetal life, the circulation is in parallel with equal right and left ventricular pressures, right ventricular (RV) predominance, and half of the cardiac output directed to the placenta circulation for its low vascular resistance. After cord clamping and initiation of ventilation, systemic vascular resistance (SVR) rises, and pulmonary vascular resistance (PVR) falls with rising in left atrial (LA) pressure and closure of the foramen oval (FO), and transition to a series circulation, finalized after several days by closure of the ductus arteriosus (through increase in oxygen tension, endothelin I and catechol amines and reduction in prostaglandins). Early infections during this transition period can interfere with this process leading to PPHN, right to left shunt via FO and persistent ductus arteriosus (PDA) (25).

During sepsis, the difference between neonates, children, and adults have also been described at the level of endothelium and interaction between pathogen and immune system (28). Differently from adults, neonates have reduced recruitment of polymorphonucleate cells (PMN) to the site of infection, diminished phagocytic activity, and intracellular killing by reactive oxygen radicals, and a higher pathogen load per ml of blood as part as a “microbe-tolerant” strategy (29, 30). Furthermore, *in vitro* studies showed lower levels of circulating TNF, IL-1B, and IL10 (31).

## Clinical Features of Sepsis in Neonates vs. Children and Adolescents

During the neonatal period, depending on gestation age, the timing of infection (early vs. late-onset), etiology (bacterial, fungal, or viral), and primary focus (pneumonia vs. systemic), sepsis can present with different clinical features of cardiovascular disturbance.

Lack of transition from fetal to neonatal circulation with severe PPHN and persistent fetal circulation (PFC) is a frequent complication of early-onset sepsis. Therapeutic target remains agents that act on reduction of PVR and RV support.

Late-onset sepsis can have the same clinical features of early onset sepsis or can present with increased SVR and severely reduced left ventricular (LV) function and cardiac output, disseminated intravascular coagulation (DIC), and multiorgan failure (MOF). Older children with septic shock, or “cold shock,” manifest the same features of severely depressed myocardial function.

Clinical features of septic shock in adults are reduced SVR (hypotension), standard or increased cardiac index, tachycardia and increased mixed venous saturations. This clinical presentation is also described as “warm shock” or distributive shock (32, 33). This is uncommon in children and very rare in neonates due to the developmental differences mentioned above.

## Indications for ECMO in Neonatal Sepsis

From 2012 to 2017, the Extracorporeal Life Support Organization (ELSO) reports that, of all neonates receiving ECMO, in <10% the indication was sepsis (34). In neonates where sepsis presents as RV failure, pulmonary hypertension and hypoxemia, indications for initiation of mechanical support do not differ from the ones for respiratory and/or cardiovascular failure secondary to meconium aspiration, congenital diaphragmatic hernia or pneumonia: oxygenation index >40 for more than 4 h, failure to wean from 100% oxygen despite maximal medical therapy, severe hypoxic respiratory failure and pulmonary hypertension with evidence of RV and/or LV failure. On the other hand, for neonates whose sepsis presents with systemic inflammatory response (SIRS), refractory septic shock (RSS), and MOF, the only indication for mechanical support provided by the latest ESLO guidelines is “pressor resistant hypotension” (34). At the current state, there is no consensus on level of inotropic/vasoactive support, level of organ dysfunction, time frame from onset to MOF or rapidity of medical therapy escalation that should trigger ECMO initiation for neonates with RSS. Validation of the septic shock scores for pediatric RSS in the neonatal population could potentially identify in future more targeted clinical parameters [vaso-inotrope score (VIS), arterial lactate and myocardial dysfunction] on the timing of ECMO (35).

## Ecmo Modality During Neonatal Sepsis

Depending on the critical clinical features of sepsis, neonates have been supported with different modalities of ECMO.

### Veno-Venous ECMO

During veno-venous ECMO (VV-ECMO), blood is drained from the venous system [superior vena cava (SVC) or inferior vena cava (IVC)] or right atrium (RA) and returned into the venous system (SVC or IVC) or RA after carbon dioxide removal and oxygenation. Historically, this modality was deemed to provide only respiratory support although by decreasing ventilation, it can augment cardiac output by decreasing lung over-distension thus reducing PVR and increasing venous return to the LA, improve coronary blood oxygen content and LV performance, and diminish intrathoracic pressure. Neonates with severe pneumonia and sepsis, manifesting as severe PPHN, are the best candidates for VV-ECMO support. In clinical practice, there have been no predictors able to identify for which neonates VV-ECMO will provide sufficient myocardial support not to need veno-arterial support. Failing cardiovascular support with persistent acidosis, reduced lactate clearance and low mixed venous saturation (SvO2) on VV-ECMO should trigger early conversion to VA-ECMO via cannulation of the carotid artery. In an ELSO database review of ECMO in septic children, VV-ECMO was mostly used in the neonatal age (87%) compared to older children (13%) and associated with improved survival when compared to VA-ECMO (83% vs. 70%, respectively) (16). Both in adults and children supported for respiratory failure, VV-ECMO was associated with lower complications rates and improved survival (36, 37). In the 2019 report from the Karolinska Institute on ECMO for septic shock in adults, VV-ECMO was associated with reduced ECMO and hospital survival when compared to VA (60 vs. 85%, respectively); survival was also higher for adults with LV failure (90%) when compared to distributive shock (64.7%) (20). This study reinforces the concept of different outcomes for the same condition depending on clinical features and modality of support for it.

VV-ECMO has a safer profile than VA-ECMO not requiring arterial cannulation and subsequent potential risk of arterial embolic phenomena to the brain, to the splanchnic region, and to the peripheries. A major downside of veno-venous support remains the potential for recirculation and hypoxemia. Since 2009, double-lumen cannulae were introduced, and have been widely utilized, to obviate for the risk of recirculation. Despite the positive aspect of requiring single vascular access, these devices are difficult to position correctly in neonates and children, and allow partial support when compared to a two cannulae strategy. In a single center review comparing complications of dual-lumen cannula vs. a two cannulae approach, children supported with a dual lumen-cannula had increased mechanical complications on ECMO and seizure episodes (38).

### Veno-Arterial ECMO

#### Peripheral VA-ECMO

Septic neonates in whom the clinical presentation is dominated by severely depressed myocardial function with progressive left ventricular dilatation and increased systemic vascular resistance (SVR) require VA-ECMO. This modality provides the best level of cardiovascular support to the failing heart, ensuring adequate blood flow, and oxygen delivery to organs. It also allows a decrease of inotropic drugs and vasopressors with their potential complications. During peripheral VA-ECMO, blood is drained via a cannula in the SVC/RA and is returned through a cannula inserted in the carotid artery and tip positioned ideally at the junction with the aortic arch. Cerebrovascular accidents (cerebral infarction or hemorrhage) remain the more significant and severe complication. In an ELSO database review, 22% of neonates cannulated peripherally onto VA-ECMO developed a neurologic injury (39). Despite lack of evidence, carotid artery repair, and not ligation after decannulation could potentially limit long term neurological complication; many centers do this.

Left atrial (LA) dilatation due to poor myocardial function and /or myocardial stunning can delay left ventricular recovery and cause pulmonary hemorrhage; this is particularly likely in the settings of severe left ventricular failure pre-ECMO or differential ventricular function with right better than left. Signs of LA hypertension on echo, lack of native myocardial ejection, and LV dilatation should trigger ECMO flows and SVR manipulation; consideration should rapidly be given to LA decompression via percutaneous atrial septostomy, percutaneous cannulation of the LA via the Foramen Ovale or direct surgical LA cannulation. After ECMO initiation, an echo should also document the presence of a patent ductus arteriosus which might contribute to high pulmonary to systemic flow ratio (Qp:Qs), arterial diastolic steal (reverse flow in aorta during diastole) and pulmonary over circulation with increasing levels of lactate or lack of clearance, pulmonary oedema and hemorrhage, contributing to higher morbidity of this modality.

#### Central VA-ECMO

While peripheral VA-ECMO might be sufficient to ensure adequate cardio circulatory support for isolated cardiogenic or cold shock, in the presence of distributive shock or mixed shock (distributive and cardiogenic), higher ECMO flows may be required to maintain adequate end-organ oxygen delivery and function. Central cannulation is primarily utilized in the post-cardiotomy pediatric population and in ECMO centers with cardio surgical programs.

This modality is similar to what happens in a cardiac operating theater during cardiopulmonary bypass (CPB); the sternum is open, blood is drained via a large bore cannula placed directly into the RA and returned into the ascending aorta. Higher flows up to 200–250 ml/kg/min can be achieved to meet the circulatory needs during mixed cardiogenic/distributive shock. Rarely low dose vasopressors (Vasopressin or Noradrenaline infusions) might be required to achieve physiological blood pressure for age. Flows should be adjusted to guarantee the best oxygen delivery to tissue (SvO2 60–70%) and flow adequate to meet metabolic demand and clear lactate (lactate <2 mmol/L); counter to intuition, continuous infusion of systemic vasodilators (and not ECMO flow reduction) might be required to manage hypertension on this high flow. This is usual for the first 6 h of mechanical support. As time passes and organ resuscitation continues, flow reduction is possible and guided by SvO2, lactate, peripheral perfusion and individual organ function.

Left heart distention is a possible complication as for peripheral VA-ECMO, and direct drainage of LA or LV can be addressed during the cannulation process as well as the ligation of a PDA. Disadvantages remain the invasive nature of the procedure, the potential for secondary mediastinal infection and high risk of bleeding, especially in neonates with liver dysfunction, DIC, and coagulopathy. During the first 24–48 h of support aggressive blood products replacement with Platelets, Fresh Frozen Plasma (FFP), and Cryoprecipitate might be required. Chest exploration for mediastinal blood accumulation and inspection for secondary bleeding points is frequently needed and clot removal is mandatory to limit secondary fibrinolysis and consumptive coagulopathy which can worsen bleeding. Careful monitoring of inlet and outlet pressures are fundamental when high ECMO flows on centrifugal pumps are utilized, in order to avoid hemolysis or cavitation. High ECMO flows and excessively negative venous pressure (<-20 mmHg) can lead to hemolysis which is associated with increased odds of ICU and in hospital mortality (40).

### Duration of ECMO

The duration of ECMO support for sepsis is generally 4–6 days and varies on the microorganism, clinical presentation (pneumonia vs. shock), timing of ECMO and pre-existing end-organ dysfunction. In the 2012–2016 ELSO registry report, 168 neonates received ECMO for sepsis, 41 of which had primary diagnosis of pneumonia and an average duration of support of 163 h (longest duration of 1,155 h) (34). Similar duration of mechanical support is reported in other single center studies on neonates and children with sepsis (18, 41, 42). Longer duration of ECMO might be expected for neonates with chronic or pre-ECMO lung disease and/or acute post-infective lung damage with cystic transformation to limit ventilator induced lung injury (VILI). The efficacy of antimicrobial therapy, the onset of any complications on ECMO, the development of other organ failures and the neonates' lung function all contribute to prolongation of ECMO; if lung disease is prominent and ECMO is needed for another 5–7 days, then conversion from VA to VV is common. Likewise, the adequacy of ECMO support has to be reassessed during the run and consideration placed on conversion to a different cannulation strategy. Conversion from VV to VA-ECMO might be required in neonates with early PPHN/PFC and worsening signs and symptoms of cardiogenic/distributive shock. Persistent lung disease and respiratory failure after the resolution of cardiovascular instability might trigger conversion from VA to VV-ECMO. Long term VV-ECMO for 3–6 months might be required for severe lung parenchyma injury (HSV cystic pneumonia or necrotizing pneumonitis, severe VILI). An alternative cannulation modality recently deployed in our center is transthoracic RA-PA ECMO which offers reduced mechanical complications (cannula migration, skin breakdown and site infection), no recirculation and RV support. Destination of therapy might involve lung transplant or withdrawal of active treatment in the face of no lung recovery and in countries where neonatal/infant lung transplant is not undertaken.

Furthermore, increased duration of ECMO can be expected in the presence of secondary acquired infections (43, 44). This risk is higher in previously septic neonates because of the immaturity of the immune system, because of the immunosuppression due to the initial pathogen leading to ECMO and the immunomodulatory effect of the circuit itself. In a 2018 report by Cashen, although not affecting outcome, 16% of neonates and children on ECMO acquired a secondary infection at a median time of 5.2 days from initiation (45).

## Risk Factors and Outcome

ECMO for neonatal sepsis has been utilized for over three decades with variable survival rates (Table 2). An ELSO registry report from 2014 to 2019 describes 119 neonates supported for sepsis with a survival rate of 51% (12). Survival rate for older children vary from to 43 to 74% based on local experience, while reported mortality for adults remains high, up to 75% (17, 18, 21, 46) (Table 1).

**Table 2:** Neonatal reports for ECMO and sepsis.

**References**	**Study population**	**Neonates included**	**Method**	**Total survival (%)**	**Neonatal survival (%)**	**Predictors of mortality**
Meyer et al. (13)	1,060 neonates (S)	1,060 (S)	ELSO registry retrospective	77	77	CPR pre-ECMO, low pH & high ventilatory rate
Reiterer et al. (14)	43 neonates resp. failure	9 (RSS)	SCR	65	44	–
Skinner et al. (16)	4,551 children (S)	3,645 (S)	ELSO registry retrospective	68	73	VA-ECMO compared to VV-ECMO
Chang et al. (19)	55 children (RSS)	4 (RSS)	SCR	31	25	Higher SOFA score
Rambaud et al. (15)	22 children (RSS)	14 (RSS)	ELSO registry retrospective	59	64	Higher inotropic requirement pre-ECMO
Sole et al. (17)	21 children (RSS)	12 (RSS)	SCR	43	50	Disease time before ECMO

*S, sepsis; RSS, refractory septic shock; SCR, single center retrospective*.

A multitude of factors influences the outcome after ECMO. Even a simple binary measure like survival or death is dependent on underlying pathogen and natural history of the disease, on clinical presentation and timing of support, on individual risk factors (age, weight, maturity of immune system, nutritional status), on the modality of support and the onset of complications.

Underlying etiological microorganism has been described as influencing outcome.

Survival with bacterial sepsis has been reported up to 75%; mortality for candida infection pre-ECMO remains very high in neonates (61%), children (69%), and adults (81%) (47).

Only one in four neonates supported with ECMO for herpes (HSV) survived, with sepsis/septic shock independently associated with mortality (OR 10.2) (42).

Similarly, survival after adenoviral infection for neonates supported with ECMO was only 11% (48). The high mortality for neonates on ECMO for disseminated viral infections might be correlated to the immaturity of the immune system, especially in premature babies, together with the invasive nature of the viral pathogen responsible for severe neurological and hepatic cytotoxic effect.

In a large ELSO review of 7,190 neonates supported on ECMO, the authors identified birth weight <3 Kg, gestational age <34 weeks and VA-ECMO as factors associated with neurological events, especially cerebral hemorrhage. In the same review, of 366 neonates supported for sepsis, 33% developed a neurological injury (49).

It remains difficult to ascertain if the onset of neurological events is solely due to ECMO or intrinsic to the natural history of the disease. In a retrospective cohort review of neonates with bacteremia, neurological complications were present in 19.4% of the study population who presented with septic shock and associated with a 57.1% mortality (50).

Similarly, intraventricular hemorrhage (IVH) grade III and IV were reported in 18% of premature babies with antenatal infection compared to 8.6% without infection (51).

Over the last two decades, as ECMO deployment has become standard of care for tertiary units, outcomes have improved, and complications have diminished, eligibility criteria have become less rigid. Gestational age lower than 34 weeks and birth weight under 2 Kg have become relative contraindications, while IVH grade III or IV and lethal chromosomal abnormality remained absolute contraindications. No significant difference in mortality for neonatal sepsis on ECMO were described for gestational age of 34 weeks (41%) and 29–33 weeks GA (46%) (52).

Timing of mechanical support for neonates with sepsis remains difficult to establish, but one can postulate that early reversal of tissue oxygen debt and organ dysfunction would improve outcome. While no data are available in neonates, in an adult case series by Cheng, ECMO for sepsis within 96 h from admission was associated with better survival when compared to later support (60 vs. 19%), reflecting an earlier reversal of multiorgan dysfunction (53). Similarly, for septic adults, persistence of shock beyond 30.5 h before ECMO initiation was associated with no survival, accentuating the importance of timing of reversal of cardiovascular dysfunction (22). Higher lactate pre-cannulation, a pH <7.2, higher VIS and the presence of a cardiac arrest pre-ECMO have been identified as predictors for increased ECMO mortality (19, 54).

While ECMO remains a supportive therapy, administration of adequate antimicrobial therapy remains fundamental to improve survival of septic neonates. Vast interest in ECMO research, for the last decade, has been the pharmacokinetic (PK) and the pharmacodynamics (PD) of medications, especially of antimicrobials, during ECLS. Neonates have an immature liver and kidney and their capacity of metabolizing and excreting drugs varies between individuals. Septic neonates represent even more a complex system because of fluid overload and changes in distribution volume (Vd), multiorgan dysfunction (kidneys and liver), hypoalbuminemia, capillary leak, and disruption in perfusion. Adding an ECMO circuit to the equation modifies further the Vd and allows for medications to be sequestered within its component (both polyvinylchloride of tubing and poly-methyl pentene of the oxygenator). Careful dosing and monitoring of the efficacy of antimicrobial therapy is fundamental for successful weaning off mechanical support. In a recent review, Raffaeli gives great insight in the pharmacotherapy of antimicrobials, sedatives and inotropic drugs for neonates supported with ECMO (55).

## Future Directions

### Earlier Use of ECMO With Better Technology and Less Sick Patients

Survival and long-term sequelae post-ECMO are multifactorial, only some of which are modifiable when a septic neonate is referred for ECMO. Early referral and/or transfer to an ECMO center might reduce the time neonates are exposed to high ventilatory pressures, and limit potential VILI, and excessive inotropic and vasopressor support. Established end-organ dysfunction pre-ECMO might not allow the deployment of “safer” ECMO modalities (VV vs. VA-ECMO) and could increase the complication rates on ECMO (bleeding and/or thrombosis, renal dysfunction, fluid overload, and need for RRT, high blood products requirements, neurological accidents). An ELSO review by Polito identified cardiac arrest and lower pH at ECMO initiation risk factors for neurological complications in neonates (cerebral hemorrhage, infarction, seizures, and brain death) (49). Several other pediatric series highlight the weight of pre-ECMO lactate on ECMO survival (18, 56, 57). Close collaboration with neonatologists and open discussion about local protocols and indications/inclusion criteria for ECMO could reduce the delay between clinical presentation and ECMO cannulation, and therefore improve outcomes.

### Role of Adjunctive Extracorporeal Therapies

Despite limited data specific for the neonatal septic population, utilization of adjunctive extracorporeal techniques during ECMO has become standard practice. In a retrospective analysis of pediatric admissions for sepsis between 2004 and 2012 collected via Pediatric Health Information System (PHIS), 169 children under 1 year of age received both ECMO and renal replacement therapy (RRT) (58). Hypoxia, arterial hypotension, low cardiac output state, diastolic steal via PDA, and high vasoactive support can lead to acute kidney injury (AKI) in neonates with sepsis (59). In a mixed pediatric and neonatal report, the incidence of AKI on ECMO was of 60–74% while in a retrospective cohort study on neonates on ECMO, the presence of AKI was associated with a 3.2 Odds Ratio of death (60, 61). Renal replacement therapy (RRT) is commonly used in patients on ECMO to obviate for fluid overload and AKI, both associated with increased duration of circulatory support and mortality (60, 61). Furthermore, both *in vitro* and animal studies, have shown reduction of inflammatory mediators in sepsis and post cardio-pulmonary bypass (62, 63). In a case series by Blijdorp, the deployment of RRT in neonates on ECMO was associated with reduced duration of ECMO and ventilatory support (64).

Although the deployment and timing of RRT during ECMO remains controversial with non-neonatal studies showing either increased mortality or unaffected outcome, physiologically early renal support seems the best approach to reverse fluid overload and/or maintain even fluid balance, correct electrolyte disequilibrium, remove cytokines and enhance caloric intake (58, 61, 65, 66).

Thrombocytopenia-associated multi-organ failure (TAMOF) secondary to sepsis has been focus of interest over the last 5 years and has triggered utilization of plasma exchange (PE) techniques for patients off and on ECMO. In a clinical picture similar to thrombotic thrombocytopenic purpura (TTP), inflammatory mediators during sepsis can inhibit or inactivate ADAMTS-13, a metalloprotease whose deficiency leads to microangiopathic thrombosis, end-organ dysfunction and death. Children with TAMOF receiving PE have shown improved 28-days survival and reduction in end-organ dysfunction (67). In a case series of 14 children on ECMO for sepsis-related MOF and TAMOF, utilization of PE was associated with reduced organ failure index and VIS with a survival of 71.4% (68). Despite promising results of this technique in this subgroup of children, routine deployment should be balanced with the potential complications caused by it (hypotension, hypocalcaemia, coagulopathy, removal of protein-bound medications and antibiotics) (69).

Limited experience based on single case reports is available for extracorporeal blood purification techniques (EBPTs). Adsorptive therapies have been utilized mostly for hyperinflammatory syndromes (hemophagocytic lymphohystiocitosis, macrophage activation syndrome) or toxin-mediated infections manifesting as SIRS/septic shock. Two clinical reports from Japan on non ECMO neonates with sepsis/toxic shock highlight the potential adoption of these techniques on ECMO (70, 71). Similarly, non ECMO studies on adults with septic shock treated with HA330 adsorption cylinder showed improved hemodynamic parameters and decreased mortality (72).

### Adequacy of ECMO Blood Flow

In a multicenter study of children supported with ECMO for septic shock, VA-ECMO showed beneficial effect for children with a cardiac arrest and ECMO flows over 150 ml/kg/min were associated with higher survival compared to “standard” flows (survival 82 vs. 43%) (56).

### Anticoagulation Is More Complex Than in Children/Adolescents

Anticoagulation for neonates on ECMO is difficult because of the immaturity of their clotting pathways and their hypercoagulable state, and propensity for cerebral hemorrhage. In a large prospective observational cohort study, bleeding and thrombotic events in neonates receiving respiratory ECMO were 60% and 43% respectively; furthermore, 22% of the events were cerebral hemorrhage while 3.3% were intracranial infarctions (73). Bleeding and thrombotic events are responsible for increased morbidity and mortality on ECMO (73–75).

Neonates present a unique and very fine balance between anticoagulant and procoagulant state. Neonatal platelet activity is reduced compared to children and adults compensating the increased level of von Willebrand factor. Plasma levels of clotting factors are reduced while there is reduced expression of anticoagulant factors (protein C and S and antithrombin III) (76). Sepsis with DIC and thrombocytopenia puts neonates at very high risk for both thrombotic and hemorrhagic complications and adds further complexities to the anticoagulation regimen.

Precipitant that disrupt the fine neonatal hemostatic homeostasis like sepsis and/or ECMO lead to platelet activation and secondary depletion, complement cascade activation, leukocyte margination and cytokine release, thrombin generation and subsequently secondary fibrinolysis are responsible for both hemorrhagic and/or thrombotic events and the difficulty of managing anticoagulation on ECMO. In a neonatal ECMO report by Doymaz, low fibrinogen (<150 mg/dL) and low platelet count (<50.000/μL) were associated with increased risk for intracranial hemorrhage (77).

Inconsistency of blood test results due to the small volumes of blood and reagents make goal setting for anticoagulation difficult and varied throughout the world. This is particularly relevant to APTT and ACT but less so with INR and Anti-Xa. Bedside tests such as TEG or Rotem are increasingly being evaluated. Exposure to the foreign surface of the ECMO circuits only accentuates the existing coagulopathy with further consumption of clotting factors and platelets (78, 79). Future biocompatible materials might limit the need for systemic anticoagulation or antiplatelet agents on ECMO limiting transfusion requirements and hemorrhagic or thrombotic events. Circuits that act as nitric oxide donors have been investigated for this purpose for the last decade (80–82).

## Conclusions

High survival rates can be achieved for neonates with bacterial sepsis and septic shock and ECMO should be always considered in the absence of severe intracerebral pathology. Worse outcome is associated with non-bacterial sepsis, extreme prematurity, need for ECPR, higher lactate and severity of organ dysfunction pre-ECMO. Predominant pathophysiological features should dictate modality of support (VV-ECMO for right ventricular failure and PPHN, VA-ECMO for left ventricular failure and RSS). Future study is warranted to determine the optimal timing of support.

## Author Contributions

WB and RC contributed in equal parts at the development of the manuscript.

### Conflict of Interest

The authors declare that the research was conducted in the absence of any commercial or financial relationships that could be construed as a potential conflict of interest.
